# Mediators of the association between depression and migraine: a mendelian randomization study

**DOI:** 10.3389/fgene.2024.1326817

**Published:** 2024-05-31

**Authors:** Yang Li, Ge Luo, Liwang Zhou, Xuena Wang, Hui Liu, Yang Zhang, Min Yan

**Affiliations:** Department of Anesthesiology, Second Affiliated Hospital, Zhejiang University School of Medicine, Hangzhou, China

**Keywords:** causality, depression, mendelian randomization analysis, migraine disorders, risk factors

## Abstract

**Background:**

An association between depression and migraine has been reported in observational studies; however, conventional observational studies are prone to bias. This study aims to investigate the causal relationship between depression and migraine and to quantify the mediating effects of known risk factors.

**Methods:**

We applied two-sample Mendelian randomization and utilized single nucleotide polymorphisms as genetic instruments for exposure (depression) and mediators (sleep traits). We utilized summary data on genome-wide association studies for depression, sleep-related traits mediators and migraine. For depression, genome-wide association studies (depression) were utilized as a test cohort for the primary analysis. Moreover, genome-wide association studies (major depressive disorder) were utilized to test the stability of the results for the validation cohort. IVW and MR-Egger regression were applied to test the heterogeneity, and Cochran’s Q statistics were calculated to quantitatively evaluate the heterogeneity. MR-PRESSO analyses were utilized to examine and correct possible horizontal pleiotropy through removing outliers, and leave-one-out analyses were utilized to identify outlier SNPs.

**Results:**

Genetically predicted depression was associated with migraine (OR = 1.321, 95% CI: 1.184–1.473, *p* < 0.001). Furthermore, risk factors insomnia was associated with migraine risk (OR = 1.766, 95% CI: 1.120–2.784, *p* = 0.014). The mediator insomnia accounted for 19.5% of the total effect of depression on migraine.

**Conclusion:**

These results support a potential causal effect of depression on migraine, partly mediated by insomnia. Therefore, the enhancement of sleep quality and difficulty in falling asleep may reduce the migraine burden occasioned by depression.

## Background

Migraine is a common, chronic, disorder that is typically characterized by recurrent attacks of headache and accompanying symptoms such as nausea, vomiting, photophobia, and phonophobia (Headache Classification Committee of the International Headache Society IHS, 2018). In the general population, migraine is more prevalent in women than men, and the cumulative risk attains 33% and 18%, respectively. Migraine, which is one of the major neurological disorders, imposes a heavy burden on individuals and society (GBD, 2016 Neurology Collaborators, 2018). Based on the Global Burden of Disease 2010 study, migraine accounts for approximately 1% of global disability adjusted life years, and it is the 30th leading cause of disability adjusted life years (Murray et al., 2012). The annual direct and indirect costs of migraines are approximately 20 billion dollars in the United States (Stewart et al., 2003) and 27 billion euros in Europe (Stovner et al., 2008).

Depression is an often overlooked and highly prevalent psychiatric comorbidity associated with migraine (Jette et al., 2008). The association between depression and migraine has been extensively studied using observational study designs. A cross-sectional study indicated that migraine patients with psychiatric comorbidity exhibit higher healthcare utilisation propensities than migraine patients without psychiatric comorbidity (Minen and Tanev, 2014). If left untreated, the psychiatric disorder increases the risk of migraine chronification and migraine-related disability, reduces quality of life, and negatively affects treatment outcomes. An observational study indicated that the lifetime depression prevalence in persons with migraine was about three times higher than in persons with no history of migraine (Breslau et al., 2000). A meta-analysis of data obtained from 12 studies on migraine and depression noted that the prevalence estimates of depression in migraineurs is highly variable, ranging from 8.6% to 47.9% (Antonaci et al., 2011). Furthermore, migraineurs with depression are more likely to be resistant to migraine treatment and to suffer from drug overuse and disability (Peck et al., 2015). However, there is no evidence to support the following assumption: improving depression control can control migraines. In addition, there was a significant bidirectional relationship between depression and migraine (Breslau et al., 2000). This association is likely mediated by risk factors sleep traits (Rains and Poceta, 2006; Ødegård et al., 2010). Knowledge of mediation pertaining to the depression–migraine relationship will inform public health policies, such as setting priority targets for intervention to reduce the excessive risk of migraine occasioned by depression. Currently, the knowledge of mediating pathways is predominantly based on conventional observational studies that are sensitive to residual confounding and reverse causation (Smith and Ebrahim, 2003), which hamper conclusions on whether depression is more likely to cause migraine. Therefore, it is difficult to evaluate the association between depression and migraine, and their intermediates are confounded or influenced by reverse causation.

Mendelian randomization (MR) is a more robust method for causal inference than conventional observational studies, and this statement can be rationalized by Mendel’s laws and the fact that genotypes of germline genetic variation are defined at conception and are generally not associated with conventional confounders of observational studies (Smith and Ebrahim, 2003; Davey Smith, 2011). Using MR principles, causality between an exposure (depression) and an outcome (migraine) can be tested by using genetic markers associated with the exposure (Burgess et al., 2013). Genetic variants can be utilized as instrumental variables of exposures in an MR design; thus, the inference in observational studies can be enhanced. Therefore, we conducted an MR analysis to explore the causal relationship between depression and migraine.

Herein, we aimed to obtain causal estimates of the depression–migraine relationship, and to characterise the causal structure by assessing mediation effects. Because treating depression may reduce migraine symptoms, obtaining information on whether this association is causal may be of clinical interest.

## Materials and methods

### Overall study design

This study utilized a two-step MR analysis of genetic summary data to investigate the extent to which sleep traits, such as insomnia, sleep duration, daytime sleepiness, napping, and short sleep duration, explain the detrimental effect of depression on migraine risk.

The genome-wide association study summary data utilized herein are publicly available, and ethical approval and informed consent were obtained in each original study. Table 1 depicts the datasets that we included.

**TABLE 1:** Characteristics of GWAS summary statistics.

Traits	Study	Consortium	Population	Cases	Controls	Total
Depression	Howaord, et al.	PGC and UK Biobank	European	170,756	329,443	500,199
Major depressive disorder	Wray, et al.	PGC	European	135,458	344,901	480,359
Insomnia	Lane, et al.	UK Biobank	European	129,270	345,022	474,292
Sleep duration	Dashti, et al.	UK Biobank	European	—	—	446,118
Short sleep duration	Dashti, et al.	UK Biobank	European	106,192	305,742	411,934
Daytime sleepiness	Wang, et al.	UK Biobank	European	—	—	4,52,071
Napping	Dashti, et al.	UK Biobank	European	—	—	4,52,633
Migraine	Hautakangas, et al.	IHGC	European	102,084	771,257	873,341

GWAS: Genome-wide association study. PGC, psychiatric genomics consortium; IHGC, international headache genetics consortium.

### Instrument selection

**FIGURE 1:**
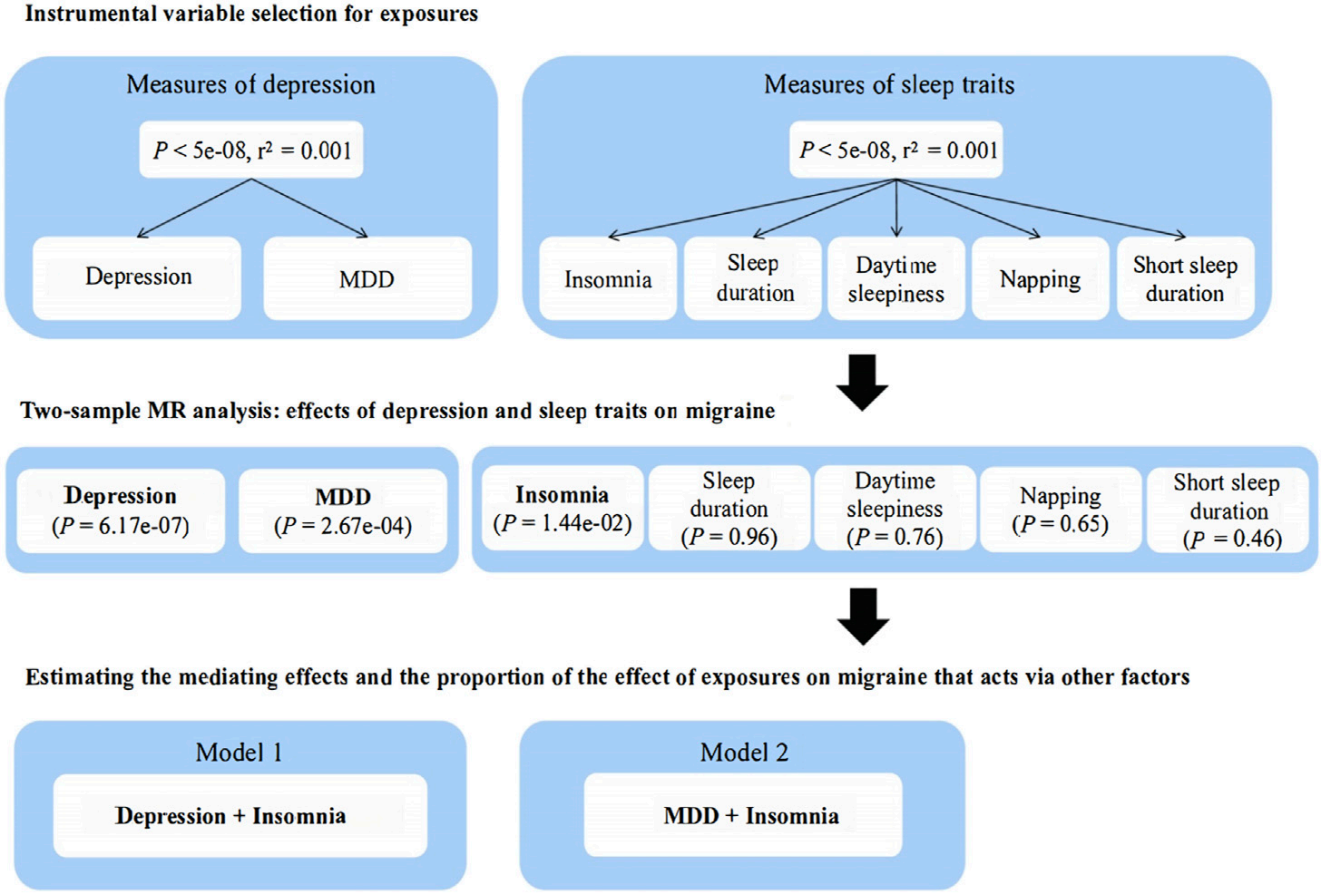
Schematic representation of this Mendelian randomization analysis. MR, Mendelian randomization; MDD, major depressive disorder.

All selected single nucleotide polymorphisms and their associations with depression, mediators, and migraine were extracted from the genome-wide association studies depicted in Supplementary Table S1. Researchers screened out the eligible genetic variants that met the conditions based on strict quality control from the genome-wide association study summary statistics of depression and various sleep-related traits including insomnia, sleep duration, daytime sleepiness, napping, and short sleep duration. A schematic overview of the present study design is illustrated in Figure 1.

When performing MR analysis using genetic variants as instrumental variables, MR analysis should be based on three principal assumptions (Boef et al., 2015; Headache Classification Committee of the International Headache Society IHS, 2018) genetic variants should be associated with the exposure; (GBD, 2016 Neurology Collaborators, 2018); genetic variants should be associated with the outcome exclusively through the exposure; and (Murray et al., 2012) genetic variants should be independent of any measured and unmeasured confounders. The single nucleotide polymorphisms associated with depressive phenotype and five sleep-related traits with genome-wide significance (*P* < 5e-8) were extracted. Because the existence of linkage disequilibrium may lead to corresponding bias, controlling linkage disequilibrium before subsequent analysis was necessary. Herein, independent single nucleotide polymorphisms were selected by setting r2 < 0.001 and window size = 10,000 kb. To further explore whether genetic variants were interfered by other confounding factors, we searched all the selected instrumental variants in Phenoscanner database (http://www.phenoscanner.medschl.cam.ac.uk), which provides detailed information pertaining to human genotype-phenotypes (Supplementary Table S2).

### Data sources

Genetic associations for depression, such as depression (*n* = 500,199 individuals) and major depressive disorder (*n* = 480,359 individuals) (Wray et al., 2018), were obtained from genome-wide association study summary statistics in psychiatric genomics consortium and UK Biobank participants of European ancestry (Table 1).

We considered a genome-wide association study for five sleep-related traits ascertained in UK Biobank: insomnia (Lane et al., 2019) (*n* = 474,292 individuals), sleep duration (Dashti et al., 2019) (*n* = 446,118 individuals), short sleep duration (*n* = 411,934 individuals), daytime sleepiness (Wang et al., 2019) (*n* = 452,071 individuals), and napping (Dashti et al., 2021) (*n* = 452,633 individuals), as depicted in Table 1. We selected all available sleep traits to provide an unbiased investigation (Luo et al., 2022). It has been indicated that the questions utilized to define patient self-reported insomnia symptoms in UK Biobank exhibit sensitivity and specificity for clinically diagnosed insomnia disorders (Jansen et al., 2019). Although daytime sleepiness is usually studied as an exposure or an outcome, herein, it was included as a mediator because the genetic structure of daytime sleepiness indicates that this trait may partly reflect sleep fragmentation (Kim et al., 2016; Daghlas et al., 2020). Genetic variants associated with sleep traits in these genome-wide association studies were also strongly correlated with corresponding objective sleep indicators (Jones et al., 2019).

We acquisitioned genetic associations with migraine from the largest meta-analysis of genome-wide association migraine studies conducted by the International Headache Genetics Consortium (Hautakangas et al., 2022). This study comprised 873,341 individuals of European ancestry (102,084 cases and 771,257 controls) from five study collections, such as IHGC 2016 (Gormley et al., 2016), 23andMe (23andMe.com), UK Biobank (ukbiobank.ac.uk), GeneRISK (generisk.fi), and Nord-Trøndelag Health Study (ntnu.edu/hunt). The characteristics of each contribution cohort have been described in previous studies. Migraine cases were defined using a range of different methods, including self-reporting, questionnaires assessing diagnostic criteria, and diagnosis by trained clinicians. All participants exhibited genetically verified European ancestry.

### Mendelian randomization analyses

With regard to depression and migraine, after harmonization of the effect alleles across the genome-wide association studies, we utilized MR analyses to determine depression-based MR estimates for migraine, which assumes the absence of invalid genetic instruments (Burgess et al., 2013), including inverse-variance weighted (IVW), MR-Egger, weighted median, maximum likelihood (ML), and penalized weighted median. The primary MR analyses were conducted using IVW regression analysis. When the MR assumptions are met, this odds ratio (OR) is an estimate of the causal effect of the exposure on outcome. All statistical analyses were performed using the R programming language (version 4.0.5), and MR analyses were conducted using the R-based package “TwoSampleMR” (version 0.5.6).

The IVW method provides the most precise estimates; however, it is sensitive to invalid instrumental variables and pleiotropy (Burgess et al., 2017). Therefore, we utilized the MR-Egger, weighted median, ML, and penalized weighted median as sensitivity analyses. The MR-Egger regression can detect possible pleiotropic effects and provide estimates after pleiotropy correction, albeit with low power (Bowden et al., 2015). The weighted median method can produce consistent causal estimates, assuming >50% of the instrumental variables from valid single nucleotide polymorphisms (Bowden et al., 2016). ML and penalized weighted median were mainly utilized to assess the robustness of MR results (Hartwig et al., 2017).

### Mediation analysis

To estimate the indirect effect of depression on migraine through sleep traits, we performed a mediation analysis that included the causal estimates from two-step MR analyses and the total effect from univariable MR analyses of depression on migraine.

First, univariate MR analysis was performed to indicate the causal effect of depression on migraine, and it was considered to be the total effect of exposure (depression) on outcome (migraine), which was estimated to be β1; by contrast, the causal estimates of depression on risk factors related to sleep traits and risk factors on migraine were β2 and β3, respectively. The indirect effects of risk factors related to sleep traits were β2*β3, which was calculated using the coefficient product method. The standard error and the confidence interval of the indirect effect were calculated using the Delta method. Second, the direct effect was the causal estimates after excluding the indirect effect, which was β1-β2*β3 (Relton and Davey Smith, 2012). Finally, to verify the potential mechanism of risk factors, we established a causal association in each univariable MR analysis. It is important to note that the estimate of each causal effect should satisfy the following relationship: |β2*β3| < |β1|. If β2*β3 = β1, the exposure of interest completely affected the outcome through mediator, and it was considered to be a complete mediator. By contrast, |β2*β3| > β1 indicated that there may be a logical error.

### Sensitivity analyses

IVW and MR-Egger regression were applied to test the heterogeneity, and Cochran’s Q statistics were calculated to quantitatively evaluate the heterogeneity. If the heterogeneity existed (*p* < 0.05), then the random effect IVW results were dominant; otherwise, it referred to the results of fixed effect IVW. MR-Egger intercept tests were performed to detect horizontal pleiotropy, and single nucleotide polymorphism and leave-one-out analyses were utilized to identify outlier single nucleotide polymorphisms driving relationships. Variance (R2) in the MR study refers to the proportion of total variation in the exposure that is explained by the genetic instruments. R2 for each trait were derived from the original study. To ensure that MR estimates minimize potential weak instrument bias, we considered a ≥ 10 *F*-statistic as sufficient for performing an MR analysis.

MR-Pleiotropy Residual Sum and Outlier methods (MR-PRESSO) analyses, as installed in the R-based package “MRPRESSO” (version 1.0), were also utilized to examine and correct potential horizontal pleiotropy through removing outliers (Ong and MacGregor, 2019), of which the distributions number was set to 1,000.

## Results

### Genetic instruments selection

Summary information of instruments identified for depression and sleep traits are presented in Supplementary Tables S1, S2. Supplementary Tables S3, S4 depicts the harmonising results of instrumental variables filtering in detail. Ambiguous single nucleotide polymorphisms with incompatible alleles or palindromic single nucleotide polymorphisms with ambiguous strand were removed and illustrated in Supplementary Table S4.

### Effect of depression on migraine and sleep traits

Using the 46 depression-related single nucleotide polymorphisms, we observed the existence of a potential causal effect of depression on the risk of migraine (OR = 1.321, 95% CI: 1.184–1.473, *p* < 0.001), which was depicted in Table 2; Supplementary Table S7. The F-statistic of the depression-related single nucleotide polymorphisms utilized herein was approximately 14.92. In addition, we also observed that a genetic predisposition to depression leads to a higher risk of insomnia (OR = 1.088, 95% CI: 1.053–1.124, *p* < 0.001) with an F-statistic of 15.31. In the validation cohort containing 33 major depressive disorder-related single nucleotide polymorphisms, we also noted a potential causal effect of depression on the risk of migraine (OR = 1.262, 95% CI: 1.114–1.431, *p* < 0.001), and the F-statistic was 16.45. Moreover, we observed that depression was associated with an increased risk of insomnia (OR = 1.082, 95% CI: 1.046–1.120, *p* < 0.001), and the f statistics was 16.43. The output pertaining to the heterogeneity analysis of depression-migraine and depression-insomnia indicated possible heterogeneity, and the results of the heterogeneity test are illustrated in Supplementary Table S8. The MR-Egger intercept was utilized to evaluate the horizontal pleiotropy, and the results did not exhibit any evidence of horizontal pleiotropy, as illustrated in Supplementary Table S9. With outliers removed (major depressive disorder-migraine: rs2005864; depression-insomnia: rs12919291, rs12967143, rs28541419, rs2876520, rs354155, rs4730387, rs4936276, rs754287, rs76954012; major depressive disorder-insomnia: rs12552, rs12958048, rs17727765, rs2005864, rs7430565, rs76485002) in the MR-PRESSO analysis, the estimate did not change materially after correction (Supplementary Tables S5, S6).

**TABLE 2:** Two-sample MR analysis of casual effects between Depression-Migraine, Sleep traits-Migraine and Depression-Sleep traits.

Exposures	Outcome	nSNPs	F Statistics	IVW					MR-Egger							Weighted median			Maximum likelihood			Penalised weighted median		
				Beta	OR (95%CI)	*p*‐Value	Q statistics	*p*‐Value	Beta	OR (95%CI)	*p*‐Value	Q statistics	*p*‐Value	Egger-intercept	*p*‐Value	Beta	OR (95%CI)	*p*‐Value	Beta	OR (95%CI)	*p*‐Value	Beta	OR (95%CI)	*p*‐Value
Depression	Migraine	46	14.92	0.278	1.321 (1.184, 1.473)	6.17E-07	77.29334	1.95E-03	−0.267	0.765 (0.431, 1.361)	0.37	71.48675	5.48E-03	0.01666562	6.53E-02	0.203	1.225 (1.074, 1.397)	2.51E-03	0.282	1.326 (1.217, 1.445)	1.34E-10	0.174	1.191 (1.043, 1.360)	0.01
Major depressive disorder	Migraine	33	16.45	0.233	1.262 (1.114, 1.431)	2.67E-04	64.2067	6.23E-04	0.054	1.055 (0.547, 2.037)	0.87	63.59901	4.98E-04	0.005863433	5.90E-01	0.236	1.266 (1.095, 1.464)	1.47E-03	0.237	1.268 (1.157, 1.389)	3.70E-07	0.237	1.268 (1.104, 1.456)	8.01E-04
Insomnia	Migraine	36	14.06	0.569	1.766 (1.120, 2.784)	1.44E-02	75.53694	8.38E-05	1.570	4.804 (0.641, 36.016)	0.14	73.37977	1.03E-04	−0.00929754	0.32	0.812	2.253 (1.368, 3.710)	1.41E-03	0.603	1.827 (1.325, 2.520)	2.38E-04	0.879	2.409 (1.478, 3.926)	4.16E-04
Sleep duration	Migraine	60	13.61	−0.005	0.995 (0.822, 1.204)	9.60E-01	94.14742	2.47E-03	0.615	1.850 (0.685, 4.993)	0.23	91.68991	3.17E-03	−0.009538244	0.22	0.039	1.039 (0.822, 1.315)	0.75	−0.005	0.995 (0.852, 1.161)	0.95	0.021	1.021 (0.804, 1.297)	0.86
Daytime sleepiness	Migraine	35	16.90	−0.102	0.903 (0.465, 1.754)	7.60E-01	84.71766	3.25E-06	2.053	7.790 (0.132, 458.750)	0.33	81.97749	4.75E-06	−0.01443297	0.30	−0.189	0.827 (0.418, 1.638)	0.59	−0.101	0.904 (0.584, 1.399)	0.65	−0.190	0.827 (0.417, 1.640)	0.59
Napping	Migraine	101	14.59	0.066	1.068 (0.805, 1.416)	6.50E-01	177.6876	2.82E-06	0.423	1.526 (0.341, 6.833)	0.58	177.2824	2.28E-06	−0.00296351	0.64	0.037	1.038 (0.743, 1.449)	0.83	0.068	1.070 (0.861, 1.331)	0.54	0.025	1.025 (0.736, 1.428)	0.88
Short sleep duration	Migraine	24	15.56	0.296	1.344 (0.610, 2.964)	4.60E-01	44.29675	4.84E-03	−0.414	0.661 (0.006, 78.508)	0.87	44.12155	3.42E-03	0.004401645	0.77	0.473	1.605 (0.659, 3.913)	0.30	0.313	1.368 (0.760, 2.461)	0.30	0.483	1.620 (0.684, 3.841)	0.27
Major depression	Insomnia	36	15.31	0.084	1.088 (1.053, 1.124)	3.73E-07	128.9682	1.11E-12	0.051	1.052 (0.885, 1.251)	0.57	128.4091	6.89E-13	0.001009887	0.70	0.080	1.083 (1.052, 1.115)	9.82E-08	0.094	1.098 (1.078, 1.119)	4.14E-23	0.089	1.093 (1.062, 1.124)	5.96E-10
Major depressive disorder	Insomnia	30	16.43	0.079	1.082 (1.046, 1.120)	6.73E-06	123.1707	1.42E-13	0.008	1.008 (0.859, 1.183)	0.92	119.8201	2.50E-13	0.002653386	0.38	0.054	1.055 (1.022, 1.090)	1.15E-03	0.088	1.092 (1.071, 1.112)	4.55E-20	0.050	1.051 (1.019, 1.083)	1.35E-03

MR, mendelian randomization; nSNPs, number of single nucleotide polymorphisms; IVW, inverse-variance weighted; OR, odds ratio. 95% CI, 95% confidence interval.CreditValidation Error Authors: Xuena Wang, Liwang Zhou, please check and link manually.

**FIGURE 2:**
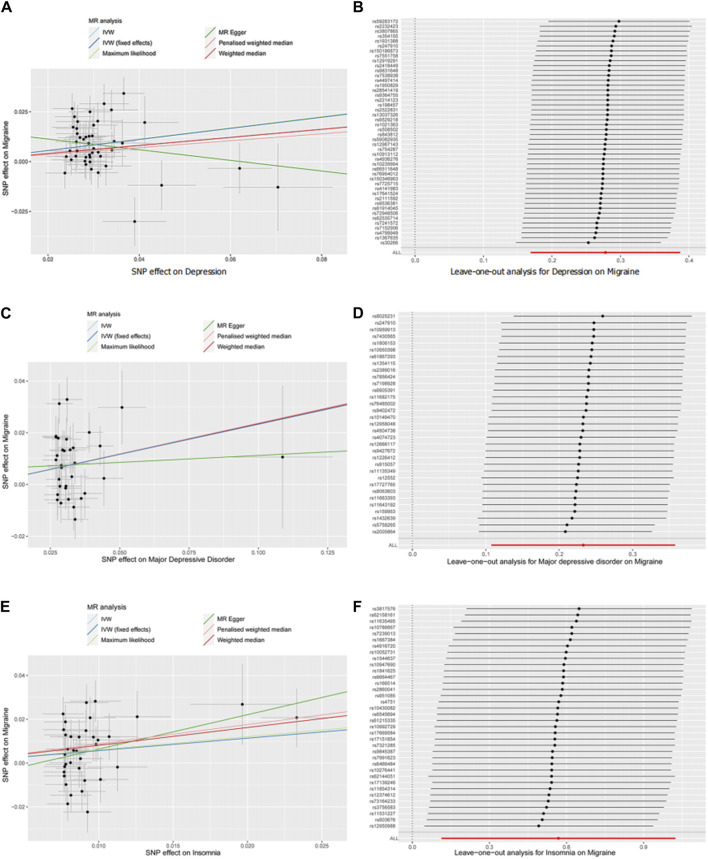
Scatter plot **(A,C,E)** and leave-one-out (**B,D,F)** analysis of two-sample Mendelian randomization studies exploring the association between interested exposures (depression, MDD, and insomnia) to migraine. MR, Mendelian randomization; SNP, single nucleotide polymorphism; IVW, inverse variant-weighted.

**FIGURE 3:**
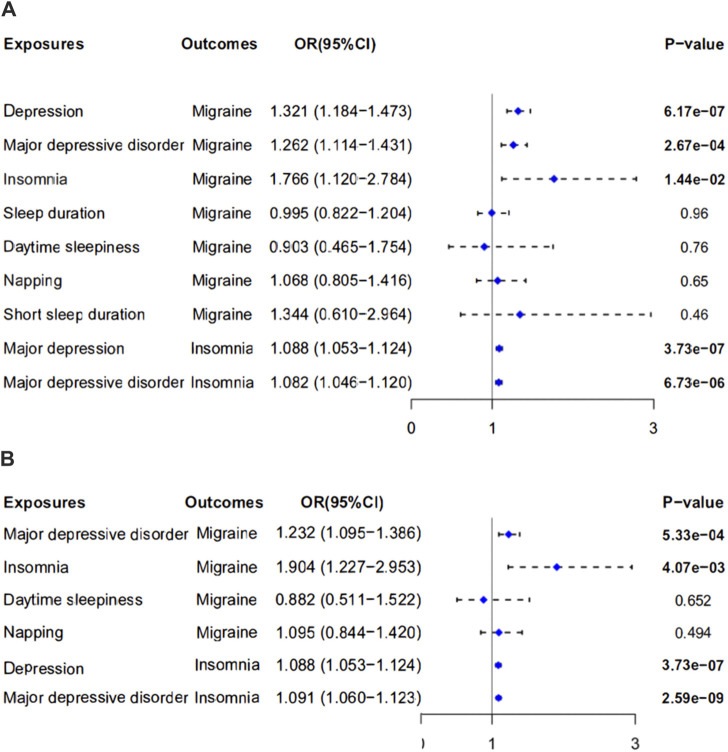
Forest plot of two-sample Mendelian randomization studies exploring associations between exposures (depression and sleep traits) to outcomes (migraine and insomnia). Outliers were reserved in plot **(A)** while eliminated in **(B)**. OR, odds ratio; 95%CI: 95% confidence interval.

Meanwhile, single nucleotide polymorphism did not affect the overall effect of depression-migraine and depression-insomnia in the leave-one-out sensitivity analysis (Figure 2B, D; Supplementary Figures S1, S2). The funnel plot was symmetrical, which indicated no pleiotropy (Supplementary Figures S3–S6). Furthermore, the scatter plot depicts the individual putative causal effect, and a significant positive correlation was observed between depression and migraine and between depression and insomnia (Figure 2A, C; Supplementary Figures S7, S8). The intercepts calculated in the MR analysis method were close to zero, which indicated that the probability of horizontal pleiotropy was low. Forest plots depicted the causal effect estimates between each single nucleotide polymorphism and the outcome, and they illustrated the combination of the effect estimates based on IVW and MR-Egger regression (Figures 3A, B; Supplementary Figures S9–S12).

### Effect of mediators on migraine

In conformance with the observations on depression, a close association between genetically predicted insomnia and migraine risk was observed, as illustrated in Table 2; Supplementary Table S7 (IVW: OR = 1.766, 95% CI: 1.120–2.784, *p* = 0.014; MR-Egger: OR = 4.804, 95% CI: 0.641–36.016, *p* = 0.14; weighted median: OR = 2.253, 95% CI: 1.368–3.710, *p* = 0.001; maximum likelihood: OR = 1.827, 95% CI: 1.325–2.520, *p* < 0.001; penalized weighted median: OR = 2.409, 95% CI; 1.478–3.926, *p* < 0.001). In the primary analyses using IVW, other genetically determined sleep traits, such as sleep duration, daytime sleepiness, napping, and short sleep duration, were not associated with the risk of migraine (Table 2; Supplementary Table S7). It was observed that single nucleotide polymorphisms identified in insomnia and migraine were available instruments, with F-statistics = 14.06. In addition, the heterogeneity was detected using MR-Egger and IVW for insomnia (MR-Egger: Q statistics = 73.380, *p* < 0.001; IVW: Q statistics = 75.537, *p* < 0.001), and MR-Egger analysis exhibited no evidence of pleiotropy (Supplementary Tables S8, S9).

In the leave-one-out analysis, we observed that the risk estimates of genetically predicted insomnia and migraine did not change substantially after excluding single nucleotide polymorphism at each period (Figure 2F). The scatter plot, funnel plot, and forest plot also exhibit similar trends to depression-migraine analysis (Figure 2E; Supplementary Figure S13; Figures 3A, B).

### Mediation effects

Using mediation analyses, the effects of depression on migraine via insomnia were quantified. The mediation effect of depression on migraine mediated by insomnia were 0.048. Insomnia accounted for 17.3% of the total effect of depression on migraine (Supplementary Table S10). Meanwhile, as a validation cohort, the mediating effect of major depressive disorder on migraine mediated by insomnia was 0.045, whereas insomnia accounted for 19.3% of the total effect of major depressive disorder on migraine.

## Discussion

Using large-scale genome-wide association studies data obtained from the psychiatric genomics consortium, UK Biobank and International Headache Genetics Consortium, we observed that depression exerted a causal, promoting effect on migraine. The findings strongly support the large body of evidence from observational studies which state that depression exerts a causal effect on the development of migraine. Observational studies are often subjected to confounding factors and reverse causation. Meta-analysis of small clinical trials are subject to publication bias; small trials with positive results are more likely to be published than those without significant results.

The results from MR analysis are less likely to be biased by confounding or reverse causation than observational epidemiological results. To correctly interpret the MR results, the following points should be considered. MR studies are dependent on certain assumptions, of which the assumption pertaining to horizontal pleiotropy is considered the most challenging (Emdin et al., 2017; Davies et al., 2018). The horizontal pleiotropic effect represents the effect of variation on multiple biological pathways, which confuses the interpretation of MR results (Davies et al., 2018). We performed sensitivity analyses to assess the(reasonableness of instrumental variable assumptions and the robustness of horizontal pleiotropy as well as outliers that might invalidate or bias MR estimates. In the leave-one-out sensitivity analysis, single nucleotide polymorphism did not affect the overall effect of depression on migraine and that of insomnia on migraine (Figures 2B, D, F). The funnel plots were symmetrical, indicating no pleiotropy (Supplementary Figures S4, S6, S13). The scatter plot exhibited a significantly positive correlation between depression and migraine and between insomnia and migraine (Figures 2A, C, E). The consistency of the estimates obtained from different approaches and analytical methods indicates strong support for the causal effect of depression on migraine.

Depression is a crucial risk factor in the development and morbidity of migraine patients. After adjusting for sociodemographic variables, depression was a significant predictor of migraine onset (OR = 1.65, 95% CI 1.12–2.45) (Ashina et al., 2012). In addition, the risk of migraine onset increased with the severity of depression. Compared to study respondents with no or mild depression, patients with moderate (OR = 1.77, 95% CI 1.25–2.52), moderately severe (OR = 2.35, 95% CI 1.53–3.62), and depression (OR = 2.53, 95% CI 1.52–4.21) exhibited an increased risk and increased migraine incidence (Bigal et al., 2008).

Additionally, the in-depth analyses apparently rationalized this causal association between depression and migraine. We first identified sleep-related phenotypes, including insomnia, sleep duration, daytime sleepiness, napping, and short sleep duration, that might exert a mediating effect that links depression with migraine incidence. Substantial evidence indicates that there is a possible association between sleep and migraine (Uhlig et al., 2014; Daghlas et al., 2020). Daghlas et al. reported that sleep disturbances might increase the risk of migraine using MR analyses (Daghlas et al., 2020). Because sleep disturbances is a well-established cause of migraine, it might act as a key intermediate factor in the depression–migraine pathway. Herein, the results, which cohere with the aforementioned finding, further implied that there is a close association between genetically predicted insomnia and migraine risk (IVW: OR = 1.766, 95% CI: 1.120–2.784, *p* = 0.014), with up to a 19.5% mediation effect of depression on migraine mediated by insomnia.

The biological, genetic, and environmental risk factors may converge to create a brain state that predisposes individuals to migraine and psychiatric comorbidities such as depression (Antonaci et al., 2011). The efficacy of tricyclic antidepressants in preventing migraine indicates that depression and migraine share a common pathogenesis (Breslau, 2002). The current hypotheses pertaining to the neurobiological mechanism of depression for migraine progression include the co-dysfunction of the serotonergic system, hormonal influences, HPA axis hyperactivity, overuse of medications due to lack of appropriate coping behaviors, central sensitivity of sensory and emotional neural networks, and inherent ideologies that amplify pain and unpleasant associated features (Baskin and Smitherman, 2009; Smitherman et al., 2011). Inflammatory markers, such as C-reactive protein, tumor necrosis factor-α, and interleukin-6, were elevated in both depression and migraine (Welch et al., 2006; Furtado and Katzman, 2015). Migraine increases allostatic load, eventually leading to adaptive dysfunction and central sensitivity. By contrast, adaptive dysfunction and central sensitivity predispose individuals to both migraine and depression. Insomnia co-exists with migraine and depression, and migraine and depression negatively impact sleep quality. Simultaneously, poor sleep quality can also predispose individuals to migraine. Therefore, we investigated the causal relationship between depression and migraine using sleep-related traits as a mediating effect, and we observed that depression exerted a stimulant effect on migraine, with insomnia acting as a mediator.

In summary, the findings supported the following hypothesis: depression increases the migraine risk. Therefore, strategies that enhance the management of psychiatric complications during migraine require considerable research attention. First, in regard to the treatment of migraine, individuals should consider the level of depression. Moreover, in regard to the occurrence of insomnia, they should focus on the patient’s sleep conditions, timely treatment, and intervention.

This study exhibited several limitations. First, most of the conducted genetic research has focused on European populations. The MR Studies included herein utilized genome-wide association study summary statistics from European ancestry cohorts such as the psychiatric genomics consortium, UK Biobank, and International Headache Genetics Consortium. Whether these results are generalizable remains unknown. To address this European bias, future genetic studies should focus on different ancestors. Second, it may be more effective to calculate estimates within subgroup analysis of potential modifiers such as sex and region, as the possibility of residual effect modifiers. Finally, the sample size for migraine subgroup analysis was relatively small. Therefore, we did not conduct subgroup analyses of migraine with aura and migraine without aura.

## Conclusion

This two-sample MR study indicated that genetically determined depression levels are associated with migraine risk. In addition, the mediating role of insomnia between depression and migraine is significant, and with regard to several sensitivity analyses, the evidence of genetic pleiotropy was minimal, which increases the robustness of the observation. Therefore, to assess the effect of depression on migraine patients and to effectively understand the biological mechanisms, appropriately designed trials are imperative.

## Data Availability

The original contributions presented in the study are included in the article/Supplementary Material, further inquiries can be directed to the corresponding author.
