# Supplementary material for: Mediators of the association between depression and migraine: a mendelian randomization study
Source: Front Genet. 2024 May 31;15:1326817. doi: 10.3389/fgene.2024.1326817 (PMC11176467; doi:10.3389/fgene.2024.1326817)
Supplement: Supplementary file 1 [file DataSheet2.DOCX]

sFigure 1. Leave-one-out analysis for depression on insomnia.

sFigure 2. Leave-one-out analysis for major depressive disorder (MDD) on insomnia.

sFigure 3. Funnel plot of depression on insomnia.

sFigure 4. Funnel plot of depression on migraine.

sFigure 5. Funnel plot of MDD on insomnia.

sFigure 6. Funnel plot of MDD on migraine.

sFigure 7. Scatter plot of depression on insomnia.

sFigure 8. Scatter plot of MDD on insomnia.

sFigure 9. Scatter plot of depression on migraine.

sFigure 10. Scatter plot of depression on insomnia.

sFigure 11. Scatter plot of MDD on migraine.

sFigure 12. Scatter plot of MDD on insomnia.

sFigure 13. Funnel plot of insomnia o migraine.
